# Efficacy of a Homemade Very Low Calorie Diet to Achieve Prevention or Remission of Type 2 Diabetes: A Pilot Study

**DOI:** 10.1111/jhn.70128

**Published:** 2025-09-25

**Authors:** Kirsty A. Hickson, Charlotte Heppenstall, Andrew Treweeke, Emma Coghill, Ian L. Megson, Sally Nicolson, David P. Macfarlane, Sandra M. MacRury

**Affiliations:** ^1^ Institute of Health Research and Innovation, Life Sciences Innovation Centre, Inverness Campus University of the Highlands and Islands Inverness Scotland; ^2^ Department of Diabetes, Highland Diabetes Centre UHI House, NHS Highland Inverness Scotland

**Keywords:** diabetes, prediabetes, very low‐calorie diet, weight loss

## Abstract

**Introduction:**

Very low calorie diets have achieved remission of type 2 diabetes in obese individuals and lowered cardiovascular risk. However, there is less evidence for prevention of diabetes with these diets. Oxidative stress is a feature of obesity, diabetes and cardiovascular disease, but it is not clear what impact these diets have on oxidative stress in diabetes or prediabetes. Commercial meal replacement products may be expensive, so in this pilot study, we sought to explore the use of a homemade very low calorie diet to achieve weight loss in obese individuals with prediabetes or early onset type 2 diabetes and the effect on oxidative stress.

**Methods:**

Individuals with a BMI > 35 kg/m^2^ and prediabetes or type 2 diabetes for less than 4 years followed a three‐phase diet plan over 48 weeks, including a homemade meal replacement of 800 calorie for the first 12 weeks. Data was collected for weight, waist circumference, glycosylated HbA1_c,_ oxidised LDL and protein carbonyls at 12 weekly intervals. Quality of Life and Self‐regulation of Eating Behaviour questionnaires were completed at 12 weekly intervals. Data was analysed using Friedman's test or mixed effects analysis for non‐normally and normally distributed data respectively.

**Results:**

Of 22 individuals enroled at baseline, 16 completed the 12 week diet intervention phase and 12 completed 48 weeks. There were significant falls in weight (*p* < 0.001), and in waist circumference (*p* < 0.001) up to 36 weeks with mean weight loss of 15 kg at 48 weeks. HbA1_c_ fell significantly (*p* < 0.001) up to 24 weeks. By 48 weeks, 67% of participants were in the prediabetes or no diabetes categories. Oxidised LDL (*p* = 0.039) and protein carbonyls (*p* = 0.005) both increased significantly through the 12 week diet intervention and the ongoing study phases. There were significant improvements in Quality of Life (*p* = 0.005) and Self‐regulation of Eating Behaviour (*p* < 0.0001) scores throughout the study.

**Conclusion:**

We have demonstrated that an inexpensive homemade very low calorie meal replacement as a weight loss tool has high levels of acceptability in addition to health and quality of life benefits in type 2 or prediabetes. The effect of very low calorie diets on inflammation and oxidative stress require further exploration.

## Summary

- Little is known about the use of hypocaloric diets, that include a total diet replacement induction, in prediabetic states or their effect on oxidative stress as a marker of cardiovascular risk.
- Ingredients for an acceptable homemade VLCD are widely available and can achieve similar benefits in terms of weight loss and cardiovascular risk to commercial products although there is a need to explore the effects on measures of oxidative stress.
- A homemade VLCD would be an inexpensive option in the management of obesity across the spectrum of dysglycaemia.

## 1 Introduction

One of the main modifiable risk factors for type 2 diabetes (T2D) is excess weight; 87% of people living with T2D aged 18‐54 years are above their ideal weight [1]. Weight loss is associated with a reduction in progression of prediabetic states, including impaired glucose tolerance, to T2D through lifestyle measures, including exercise and diet restriction [2, 3, 4]. Hypocaloric diets, associated with a structured programme and an intensive induction phase with total diet replacement, are most effective for achieving remission of T2D compared with self‐administered food‐based diets [5]. A very low calorie diet (VLCD) provides between 450 and 800 kcals daily and contains the necessary nutrients to preserve muscle mass and avoid micronutrient deficiency. Clinical support for this approach is important and following an 8–12 weeks induction phase there is a gradual structured reintroduction of food [6, 7, 8, 9]. Using a meal replacement (Counterweight plus) in individuals with T2D and a BMI > 35 kg/m^2^, the DiRECT study showed the effect of a VLCD programme on achieving remission of T2D [10]. The results showed that almost half of participants achieved remission of their T2D at 1 year, associated with around 15% reduction in body weight.

There is limited evidence on the effectiveness of lifestyle changes or dietary interventions, including VLCDs, in achieving sustained weight loss and thus continuing remission of T2D. However, rapid early weight loss does seem to be a predictor of sustained weight loss [11, 12]. There may be additional benefits, with some long‐term data in people adopting lifestyle measures suggesting an association between the magnitude of weight loss and prevention or remission of T2D, ultimately resulting in a lower subsequent incidence of cardiovascular complications [13] or a reduction in cardiovascular risk [14, 15]. VLCDs in the form of liquid meal replacement have also shown a reduction in cardiometabolic risk factors [16, 17].

Oxidative stress and low‐grade inflammation are hallmarks of obesity [18] and associated conditions [19], including T2D and cardiovascular disease [20]. Accordingly, weight loss has been shown to reduce markers of oxidative stress in people with T2D [21] and while VLCDs have been associated with reduction in oxidative stress in obesity [22], it is not clear if VLCDs impact oxidative stress specifically in individuals with T2D or prediabetes.

Commercial diet programmes such as Counterweight Plus used in DiRECT [10] are associated with significant costs to health providers or recipients. We have developed and utilised a homemade VLCD recipe according to Codex standards [23] for use within the T2D population. The ingredients of the VLCD and other foods incorporated into the diet plan are widely available in supermarkets and grocery shops and we sought to evaluate the efficacy and acceptability of a dietitian facilitated programme utilising this VLCD to prevent or remit T2D, avoiding the need for a commercial product. We also hypothesised that there would be a reduction in oxidative stress while following the VLCD in association with weight loss.

## 2 Materials and Methods

We sought to recruit 30 individuals to this pilot study to allow for attrition and ultimate participation of *n* = 20 participants. Inclusion criteria included body mass index (BMI) > 35 kg/m^2^ and a diagnosis of prediabetes (PD), (HbA1_c_ 42–47 mmol/mol) or T2D managed on diet only, mono or dual oral therapy within 4 years of diagnosis. Medication for T2D was discontinued on commencing the study protocol. Exclusion criteria included individuals diagnosed with an eating disorder or other mental health issues, pregnant or planning pregnancy, evidence of malignancy, severe heart failure or EGFR < 30 mL/min.

Participants were identified by general practitioners, practice nurses and other healthcare professionals via posters and leaflets in various health care settings in the north of Scotland The study was conducted in a hospital‐based research clinic and according to the principles of the Helsinki agreement and received NHS ethical approval; North of Scotland REC Committee − 21/NS/0055.

### 2.1 Study Procedure

The study was divided into three sections:

Phase 1 (0–12 weeks): homemade VLCD (800 kcal daily) split into three meal replacements with the addition of vegetables and multivitamin and omega 3 supplements. Diet composition is shown in Table S1 and comparison with CODEX standards in Table S2. Participants were responsible for sourcing and making all their own meal replacements. Multivitamin and omega 3 supplements were provided at the study centre.

Phase 2 (12–24 weeks): Gradual food reintroduction; one meal daily between weeks 13‐16; two meals daily between Weeks 17 and 20 and three meals daily between 20 and 24 weeks.

Phase 3 (24–48 weeks): Weight maintenance; three meals daily.

### 2.2 Data Collection

The following were collected at baseline and twelve weekly intervals up to 48 weeks: weight, height, waist circumference, and venous blood samples for HbA1_c_ (Tosoh Bioscience Inc, OH, USA), and the oxidative stress markers oxidised LDL (oxLDL), (HNE‐LDL, Human ELISA Kit, Abcam Ltd, Cambridge, UK) and protein carbonyls (Protein Carbonyl Colorimetric Assay Kit, Cayman Chemical, Ann Arbor, MI, USA).

Participants were asked to complete a Quality of Life (QOL) questionnaire (EQ‐5D) and a self‐regulation of eating questionnaire (SREBQ) at each visit in addition to a diet acceptability questionnaire 4 weeks after commencement of the VLCD phase. They were also asked to complete a 3 day food diary, as a paper record, before they commenced the study along with a record of fruit and vegetable intake throughout the 12 week intervention phase.

### 2.3 Statistical Analysis

Data were tested for normality using the D'agostino test. Non‐normally distributed data (median and 95% CI) were analysed by a repeated measures Friedman test, for comparisons of data sets across study time points, with Dunn's post‐hoc analysis. Normally distributed data (mean and standard error of the mean; SEM) were analysed using mixed effects analysis with Dunn's posttest to account for missing data points. Significance was assumed at *p* < 0.05.

## 3 Results

**Figure 1:**
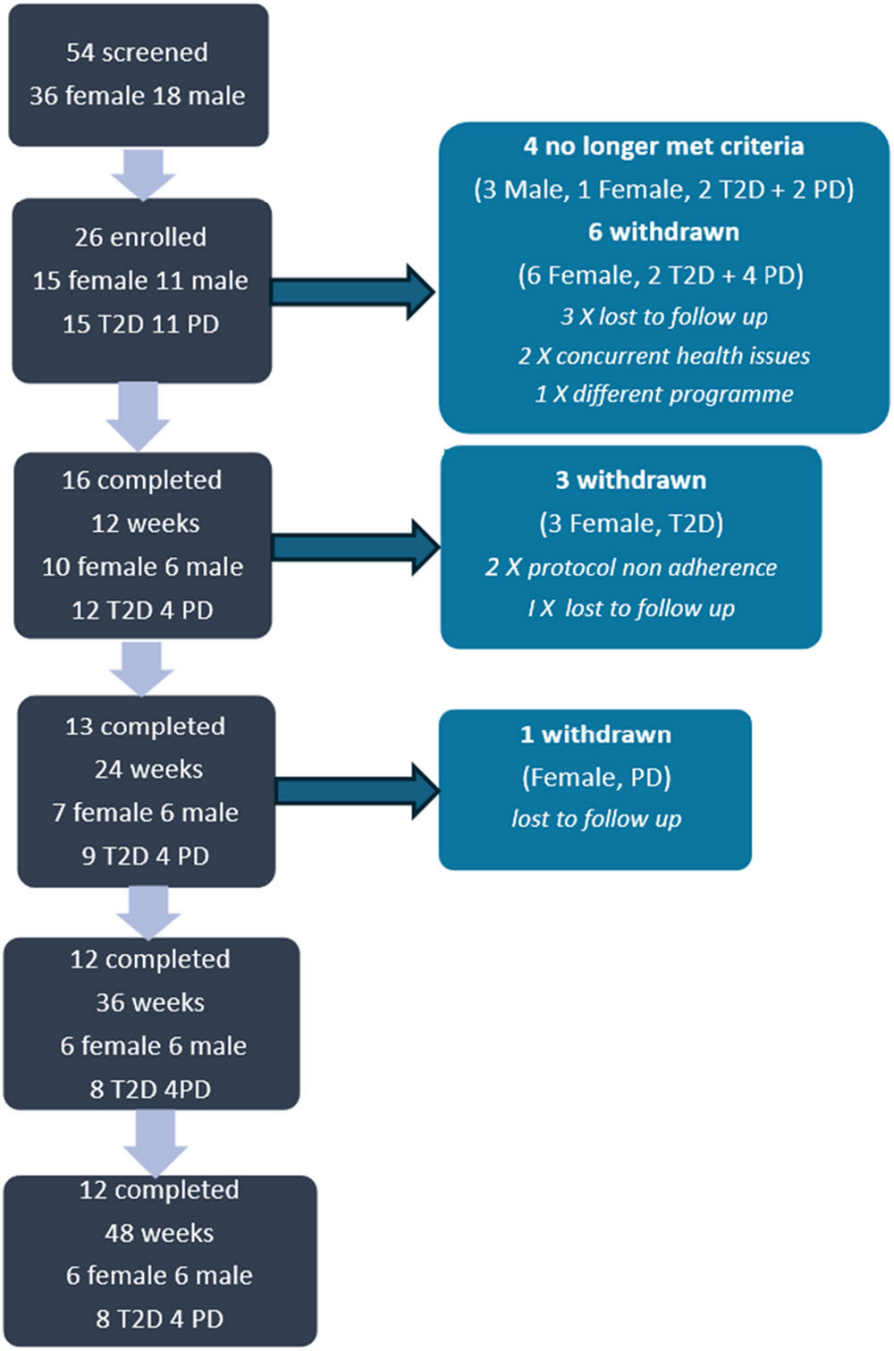
Recruitment and attrition numbers over 48 weeks of study.

A total of 54 individuals were screened, with 26 enroled. Four individuals dropped out before study commencement, with 22 completing baseline visit 0. Reasons for non‐enrolment included failure to meet study inclusion criteria, such as change in BMI or HbA1_c_ at the time of enrolment. A further eight individuals had withdrawn by week 12 (end of VLCD intervention). Thirteen individuals completed week 24 and twelve of these completed the study to 36 and 48 weeks, full details are shown in Figure 1.

### 3.1 Baseline Characteristics

Baseline characteristics of the group who completed the 12 week intervention phases of the study are shown in Table 1. Twelve of these participants had T2D diagnosed within 4 years of commencement of the study and four had prediabetes.

**Table 1:** Baseline characteristics of individuals reaching 12 weeks, *n* = 16. Data are mean ± SD.

Gender	Female *n* = 10 Male *n* = 6
Age (years)	51.7 (10.3)
Weight (kg)	125.3 (20.2)
BMI (kg/m^2^)	44.5 (7.2)
Waist circumference (cm)	135.0 (15.6)
HbA1c (mmol/mol)	56.9 (10.9)

### 3.2 Weight

**Figure 2:**
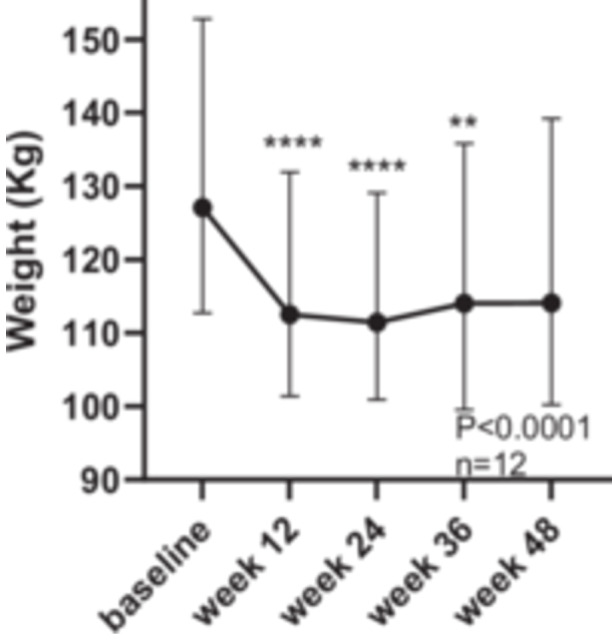
Change in weight over 48 weeks, median ± 95% CI. (**p < 0.01, ****p < 0.0001) Friedman test, with Dunn's multiple comparison test.

Median weight was significantly lower at each time point compared with baseline, with the greatest drop at 12 weeks at the end of phase 1, (127 (CI: 112.7–152.8) vs. 112.5 (CI: 101.4–131.9 kg, *p* < 0.0001, Week 12; vs. 111.4 (CI: 100.9–129.1) kg, Week 24, *p* < 0.0001; vs. 114.1 (CI 99.5–135.8) kg, Week 36, *p* < 0.01 and 114.1 (CI: 100.2–139.2) kg, Week 48, NS, Friedman test, (with Dunn's multiple comparison test), Figure 2.

### 3.3 Waist Circumference

**Figure 3:**
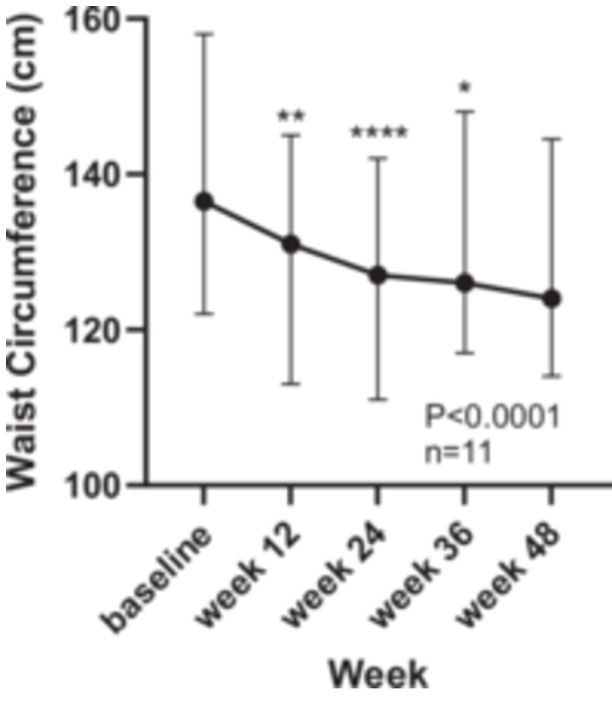
Change in waist circumference over 48 weeks, median and 95% CI. (*p < 0.05; **p < 0.01; ****p < 0.0001). Friedman repeated measures test with Dunn's multiple comparison's test.

There was a significant fall in waist circumference from baseline throughout the study, with the greatest reductions at 12 and 24 weeks (136.5 (CI: 122–158) vs. 131 (CI 113–145) cm, *p* = 0.01, 12 weeks; vs. 127 (CI: 111–142) cm, *p* < 0.001, 24 weeks; vs. 126 (CI: 148–117) cm, *p* < 0.05), Week 36 and 124 (CI: 114−144) cm, NS, Week 48, Figure 3.

### 3.4 Glycaemic Control

**Figure 4:**
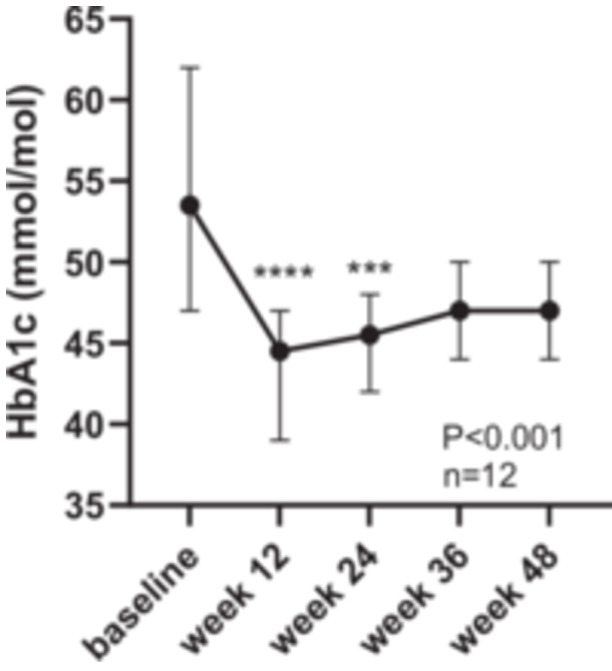
Change in HbA1c over 48 weeks, median and 95% CI. (***p < 0.001, ****p < 0.0001). Friedman test, with Dunn's multiple comparison test.

There was also a significant fall in median HbA1c from baseline at 12 weeks (53.5 (CI: 47–62) vs. 44.5 (CI: 39–47) mmol/mol, *p* < 0.0001; vs. 45.5 (CI: 42–48) mmol/mol, Week 24, *p* = 0.001), but this was no longer statistically significant at Week 36 or Week 48 (both 47 (CI: 44–50) mmol/mol), Figure 4.

### 3.5 Oxidative Stress Markers

**Figure 5:**
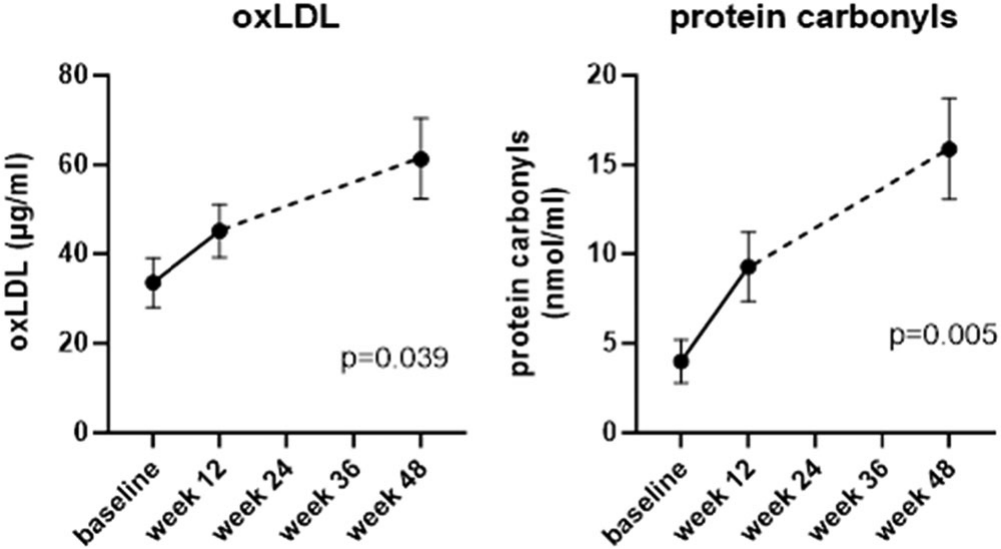
Change in oxidative stress markers over 48 weeks, mean ± SEM. (*n* = 13 for baseline and Week 12, *n* = 9 for week 48).

A mixed effect analysis was conducted on each data set on account of missing 48 week values in some instances. There was a significant, 1.5–2‐fold rise in both oxLDL and protein carbonyls at 12 weeks which was further increased at 48 weeks (oxLDL, *p* = 0.039; protein carbonyls, *p* = 0.005), Figure 5.

### 3.6 Diabetes Status

Of those with a diagnosis of T2D pre‐study who reached 12 weeks (*n* = 12), five moved into the prediabetes category (41–47 mmol/mol) and two into the ‘no diabetes’ category (< 41 mmol/mol). By 48 weeks two males (67 and 59 mmol/mol) and two females (49 and 48 mmol/mol) remained in the type 2 group. Of those in the pre‐diabetes category at the start (*n* = 4), three remained in this category and one had moved into the ‘no diabetes’ category at 12 weeks, and this remained unchanged at 48 weeks. Change in HbA1c levels along with change in weight for male and female participants over 48 weeks are shown in Table 2.

**Table 2:** Individual change in weight and HbA1c levels by gender across 48 weeks.

	Week 0	Week 12	Week 24	Week 36	Week 48
	HbA1c (mmol/mol)	HbA1c (mmol/mol)	HbA1c (mmol/mol)	HbA1c (mmol/mol)	HbA1c (mmol/mol)
	Weight (Kg)	Weight (Kg)	Weight (Kg)	Weight (Kg)	Weight (Kg)
**Male**					
1	62	39	45	50	67
	118.2	101.7	112.0	114.6	115.0
2	45	43	46	45	45
	106.5	100.7	102.9	104.2	107.0
3	44	39	41	42	40
	112.7	90.1	92.9	98.0	100.0
4	52	43	42	48	47
	152.9	131.9	129.1	135.8	140.0
5	62	48	51	60	59
	166.4	145.4	147.0	157.2	155.8
6	65	39	38	36	40
	133.3	113.2	107.5	110.0	110.6
**Female**					
1	88	66	49	50	49
	118.8	106.3	98.0	99.5	99.5
2	52	45	42	46	46
	152.8	130.8	118.6	119.6	120.8
3	57	47	48	51	50
	109.5	101.4	100.9	99.3	100.2
4	51	46	43	44	44
	130.3	118.6	21.3	124.3	126.6
5	47	44	46	46	47
	148.7	134.9	135.3	137.4	139.2
6	55	45	47	49	48
	123.8	111.8	110.8	113.5	113.2
7	63	60	67		
	114.3	108.0	113.8		
8	65	59			
	97.1	89.6			
9	55	52			
	112.0	105.8			
10	47	45			
	107.9	102.7			

### 3.7 Quality of Life

**Figure 6:**
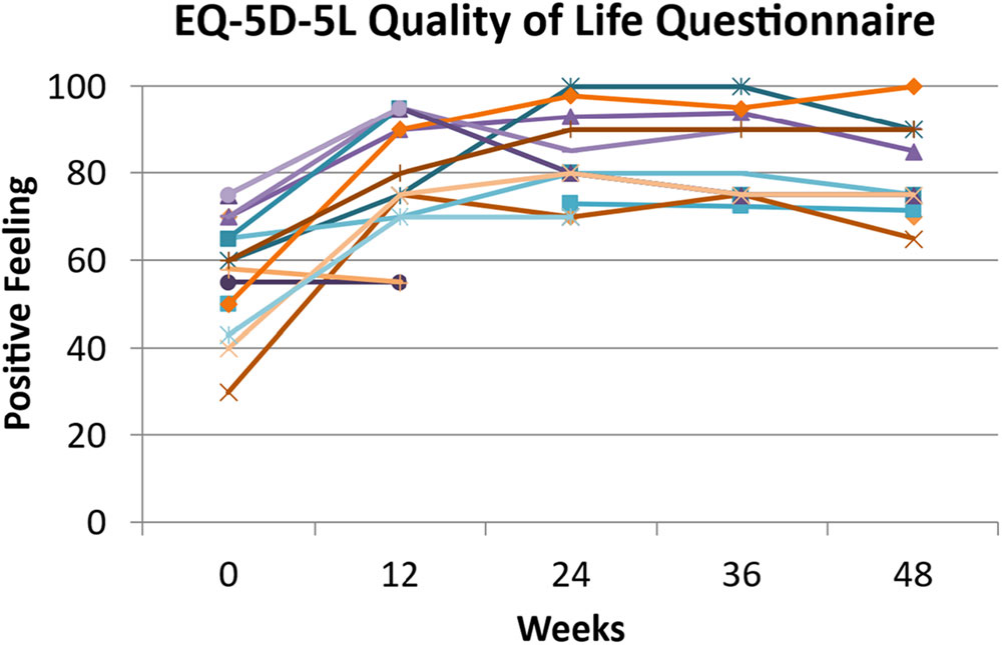
Individual QOL score (1–100) for *n* = 16 to Week 12 and *n* = 12 to Week 48.

Using the EQ‐5D‐5L Quality of life questionnaire, participants were asked on a scale from 1 to 100 to identify how they were feeling about their overall health and wellbeing. Data from participants who completed the full study are shown in Figure 6. All participants (n = 16) who completed week 12 of the study showed a marked mean increase from baseline (58.5 ± 3.3 vs. 82.5 ± 3.6, *p* < 0.001) and this was maintained throughout the study with a significantly higher average score of 80.1 ± 3.0 compared to baseline (58.7 ± 3.8) for those who completed the study at 48 weeks, *p* < 0.001.

**Figure 7:**
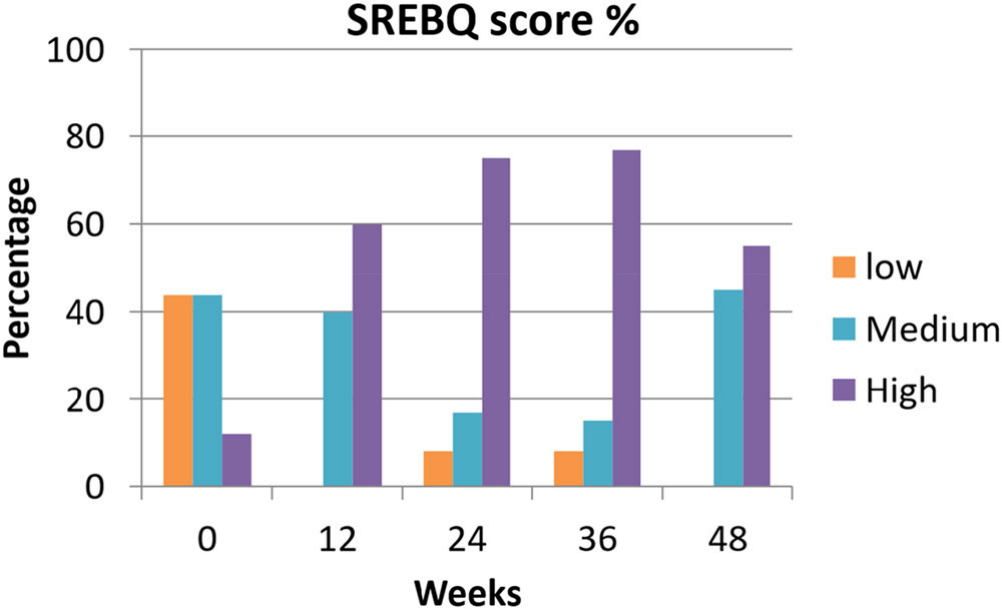
SREBQ scores for *n* = 16 to 12 weeks and for *n* = 12 to 48 weeks.

SREBQ ‐change in Self‐regulating Eating Behaviour Questionnaire Scores (Level of self‐regulatory eating skills) was highly significant across the study, chi‐square *p* < 0.0001, with 44% rating low at baseline versus 0% at week 12; 44% rating medium at baseline and vs. 40% at 12 weeks and 12% rating high baseline at baseline vs. 60% at 12 weeks, (*n* = 16). Of those who completed the study (*n* = 12), at 48 weeks, 8% rated low, 17% medium and 75% were rated high, *p* < 0.001, Figure 7.

Diet acceptability for all the participants who reached week 4 (*n* = 17) indicated that in terms of taste (94%), appearance (88%), and texture (88%) of the diet product, agreed or strongly agreed on acceptability of these factors. 82% agreed or strongly agreed it was easy to source ingredients and 94% agreed or strongly agreed that adding fruit and vegetables made it easier to stick with the diet, while 94% disagreed or strongly disagreed that the diet was expensive to follow.

### 3.8 Product Costs

Study VLCD diet average cost was £26 per week including vegetables, vitamin and omega 3 supplements. In comparison, ‘Counterweight Plus’ costs £45 per week at the noncontract product price [24], although this was likely to be slightly cheaper within an NHS contract. There is an additional training cost for healthcare professionals involved in delivering the product. There are similar NHS staff costs for supporting people undertaking both VLCD approaches.

These costs compare with the average weekly individual food costs estimated to be around £60.90 in Scotland (Office of National Statistics [25]). It has been estimated that the in‐study cost of remission of T2D in the DiRECT trial was £2359 [26]. Implementation of our diet approach in a similar environment has the potential to achieve remission costs of around 40% lower than that for DiRECT.

## 4 Discussion

In this pilot study, we demonstrated that use of a relatively inexpensive homemade VLCD in individuals with prediabetes and type 2 diabetes can achieve significant weight loss and improvements in dysglycaemia. The homemade VLCD was well tolerated, deemed inexpensive and its use, and associated dietetic support, was associated with an improvement in quality of life and self‐regulated eating behaviours.

Weight loss was comparable to other studies using commercial VLCD products and structured interventions to achieve remission of T2D such as DiRECT [10] and the NHS England type 2 path to remission study [27]. Although weight loss had plateaued by 48 weeks, no individual returned to their pre study weight and mean weight loss at this point was 15 kg compared to the mean weight loss of 10 kg at 12 months in the DiRECT study [10]

Waist circumference provides additive value as an indicator of central obesity and cardiometabolic risk [28]. Waist circumference significantly reduced over the same period of time as weight, with 5 cm at 12 weeks and an even greater change of 9.5 cm at 24 weeks.

There was a significant fall in HbA1_c_ from baseline to 24 weeks, with only two participants in the T2D range at this point (49 and 51 mmol/mol). HbA1c was lower than baseline throughout the duration of the study, although this was not statistically significant at and beyond 36 weeks due to the small study size and perhaps the inclusion of individuals with prediabetes with a lower baseline HbA1c. At the end of the main 12 week diet intervention 67% of participants had shifted to prediabetes or no diabetes and 58% were in either of these categories by the end of the study.

The prevalence of T2D is increasing globally [29] and, given the continuum of glucose intolerance and associated cardiovascular risk, it is important to identify straightforward, tolerable, economic and effective measures that can be applied in the prediabetes phase, as well the early stages of T2D, to maximise opportunities for prevention and remission of the disease, as well as reduction of diabetes‐related complications [30]. Weight loss is key to these outcomes for many people with T2D, and VLCDs within a structured programme have proven effective in achieving diabetes remission for people living with diabetes [5]. To apply this approach at scale across the continuum of T2D requires cost‐effective approaches, albeit there is little direct evidence for the use of VLCDs in prediabetes [31]. There were particular advantages to our approach using a homemade VLCD as compared to a commercial product, including the use of easily accessible inexpensive ingredients and simply prepared food. In addition, improvements in self‐regulated eating skills and quality of life scores were demonstrated over the duration of the study.

The rise in markers of oxidative stress from baseline in our study appear counterintuitive, given the reported association of obesity and hyperglycaemia with increased oxidative stress [18, 19, 20], and evidence for dietary influences and reduction in measures of oxidative stress or inflammation with balanced diets [21, 22, 32]. In particular, rapid weight loss has been associated with reduction in oxidative stress [33], as has improvement in glycaemic control in T2D with a continuous insulin infusion [34] and T2D through a variety of other therapies [35]. However, other previous studies have shown a paradoxical rise in oxidative stress markers like oxLDL with low fat diets and high vegetable intake, traditionally thought to raise antioxidant levels [36]. One explanation is that there was actually a reduction in oxLDL but reduced clearance of lipoprotein(a), creating a false elevation in measured levels [37]. It is also difficult to explain the rise in protein carbonyl levels: previous research has shown a reduction in this marker in people with T2D over a period of 6 weeks following both animal (high protein) and plant‐based diets [38]. Participants in our study had low baseline intake of fruit and vegetables, averaging 6.9 portions per week, but this increased to 28 portions per week from the start of the intervention onwards. This was biased towards fruit rather than vegetable intake and raises the question of the potential for fructose‐induced oxidative stress. Again, this is counterintuitive given the evidence for healthy ‘Mediterranean’ type diets on oxidative stress and, the natural antioxidant capacity of fruit and vegetables, although evidence for the actual antioxidant impact tends to relate to specific fruit or vegetables [39]. Moreover, most studies of the adverse effect of fructose on oxidative stress are related to animal studies and fructose‐syrup [40]. Irrespective of the interpretation of the effects of this specific VLCD on oxLDL and protein carbonyls by way of markers of oxidative stress, that both showed a significant rise lends weight to the concept that the findings are not artefactual. Whether the effects seen are specific to the dietary intervention, the markers selected (based on the impact we found with the same markers in a study of insulin pump therapy [34]) is unclear at this stage, but most evidence points to the potential for an overall fall in oxidative stress with improved glycaemic control, even if not in the acute phase of this study, when metabolism will have experienced a shock for a period of 12+ weeks and might take time to fully recover.

## 5 Limitations

Our study had a number of limitations. This was a small study with no control group and with a relatively high attrition rate (possibly influenced by the onset of the COVID19 pandemic during the study period). However, it reflected the type of approach that may be undertaken in routine clinical practice and attrition rates are known to be high in relation to VLCDs [41]. The incorporation of individuals with pre‐diabetes as well as T2D may have reduced our effect size and ability to assess for remission. Accordingly, baseline HbA1c levels were lower than in other VLCD interventions [27, 31]. The majority of participants had early T2D rather than pre‐diabetes, and it is therefore difficult to extrapolate the results, given that this subgroup is small in number, although none of the individuals with pre‐diabetes progressed to T2D and one reversed to being in the non‐diabetes category.

We only undertook an exploratory analysis of oxidative stress in our cohort. Assessment of more extensive markers of oxidative stress and/or antioxidant capacity was beyond the scope of our study but may have provided additional insights and allowed better comparisons with other published studies [20, 21, 22].

## 6 Conclusions

Given the growing incidence of obesity and the associated metabolic consequences, including the spectrum of dysglycaemia from normoglycaemia to prediabetes and diabetes, there is a need for easy to implement, cost effective, efficacious weight loss strategies to aid diabetes remission and improvements in dysglycaemia and possibly cardiovascular risk. This dietetic led pilot study has shown the acceptability and potential health and quality of life benefits of an inexpensive, homemade VLCD which merits further evaluation as a weight loss tool in those with T2D with or without obesity [27]. The effects of VLCDs on oxidative stress and inflammation should also be explored further.

## Author Contributions

**Kirsty Hickson:** conceptualisation, methodology, data curation, formal analysis, writing – review and editing. **Charlotte Heppenstall:** conceptualisation, methodology, curation of data, formal analysis, writing – review and editing. **Andrew Treweeke:** curation of data, writing – review and editing. **Emma Coghill:** curation of data, writing – review and editing. **Ian Megson:** formal analysis, supervision, writing – review and editing. **Sally Nicolson:** methodology, data curation, writing – review and editing. **David Macfarlane:** conceptualisation, methodology, formal analysis, writing – review and editing. **Sandra MacRury:** conceptualisation, methodology, data curation, formal analysis, supervision, funding acquisition, writing – original draft writing – review and editing.

## Ethics Statement

North of Scotland REC Committee − 21/NS/0055.

## Conflicts of Interest

The authors declare no conflicts of interest.

## Peer Review

The peer review history for this article is available at https://www.webofscience.com/api/gateway/wos/peer-review/10.1111/jhn.70128.

## Supporting information

Supporting Table 1.

Supporting Table 2 Very Low Calorie Diet nutrition & CODEX standards1.

## Data Availability

The data that support the findings of this study are available from the corresponding author upon reasonable request.
