# Supplementary material for: Efficacy of a Homemade Very Low Calorie Diet to Achieve Prevention or Remission of Type 2 Diabetes: A Pilot Study
Source: J Hum Nutr Diet. 2025 Sep 25;38(5):e70128. doi: 10.1111/jhn.70128 (PMC12461181; doi:10.1111/jhn.70128)
Supplement: Supplementary file 1 — Supporting Table 1. [file JHN-38-0-s002.docx]

**Supplementary Table 1**

*12 Week VLCD Phase – Daily Nutrition Plan*

**Fluids:**

- Drink 2 litres (4 pints) of water or calorie free fluids a day. You can have diet drinks and no added sugar squashes and tea and coffee with milk from milk allowance.
- Semi – skimmed/skimmed milk allowance of 100 ml per day for tea and coffee
- No alcohol during this phase as too high in calories

**Food:**

- Have 1000ml of the VLCD recipe provided below. This needs to be taken in equal part (330ml) at breakfast, lunch and evening meal. This is to start a change in habit to a regular eating pattern.

- Have 3 portions of vegetables from the list.
- Have 1 portion of fruit from the list.
- Take one multi vitamin and mineral tablet a day.

**VLCD Milk Recipe:**

- 1000 ml (1 litre) of semi skimmed milk
- 50 g skimmed milk powder (the fortified variety)
- 2-3 tbsp of milkshake syrup/powder (e.g. sugar free crusha, reduced sugar nesquick) …. …. or 100g of raspberries or 100g strawberries or 100g blackberries
- Put all ingredients in a jug and whisk with a whisk or fork. If using the berries, you may want to liquidise.
