# Supplementary material for: Efficacy of a Homemade Very Low Calorie Diet to Achieve Prevention or Remission of Type 2 Diabetes: A Pilot Study
Source: J Hum Nutr Diet. 2025 Sep 25;38(5):e70128. doi: 10.1111/jhn.70128 (PMC12461181; doi:10.1111/jhn.70128)
Supplement: Supplementary file 2 — Supporting Table 2 Very Low Calorie Diet nutrition & CODEX standards1. [file JHN-38-0-s001.doc]

**Supplementary Table 2 Homemade Very Low Calorie Diet and Codex Standards**

| **Nutrition** | **Codex Standards** | **Milk Recipe** |
| --- | --- | --- |
| **Energy (Kcals)** | 450-800 | 634 |
| **Protein (g)** | 50 | 53 |
| **Fat (g)*** |  | 17.3 |
| **Carbohydrate (g)** | 50 | 73.4 |
| **Vitamins and Minerals **** |  |  |
| *Vitamin A (µg)* | 600 | 490 |
| *Vitamin D (µg)* | 2.5 | 0.75 |
| *Vitamin E (mg)* | 10 | 0.4 |
| *Vitamin C (mg)* | 30 | 26.5 |
| *Thiamin (mg)* | 0.8 | 0.49 |
| *Riboflavin (mg)* | 1.2 | 3.1 |
| *Niacin (mg)* | 11 | 6.5 |
| *Vitamin B6 (mg)* | 2 | 0.9 |
| *Vitamin B12 (µg)* | 1 | 10.3 |
| *Folic Acid (µg)* | 200 | 105 |
| ***Calcium (mg)*** | 500 | 1840 |
| ***Phosphorus (mg)*** | 500 | 1445 |
| ***Iron (mg)*** | 16 | 0.335 |
| ***Iodine (µg)*** | 140 | 385 |
| ***Magnesium (mg)*** | 350 | 175 |
| ***Copper (mg)*** | 1.5 | TR |
| ***Zinc (mg)*** | 6 | 7 |
| ***Potassium (g)*** | 1.6 | 2.3 |
| ***Sodium (g)*** | 1 | 0.7 |

*Very low energy diets shall provide not less than 3 g of linoleic acid and less than 0.5 g a-linolenic acid. Diet supplemented with omega 3 fatty acids and linoleic acid is in milk. Both Alpha linoleic acid and linoleic acid are in the fruit, vegetables and small amounts of vegetable oil for cooking.

** Diet supplemented with vitamins and minerals to make up for short-fall.
